# Nonsurgical Tissue Repositioning: Analysis of Long-Term Results and Patient Satisfaction From 100 Absorbable Suture Suspension Cases

**DOI:** 10.1093/asjof/ojz029

**Published:** 2019-10-19

**Authors:** Julius W Few, Ginny Vachon, Stephanie Pintas, Jesse R Smith

**Affiliations:** 1 University of Chicago Pritzker School of Medicine, Chicago, IL; 2 Principal Medvantage Writing, LLC; 3 UCLA Medical School, Los Angeles, CA; 4 University of Chicago Medicine and Biological Sciences, Chicago, IL

## Abstract

**Background:**

Absorbable suspension sutures are the only nonsurgical modality approved for tissue repositioning.

**Objectives:**

To quantitate patient perceptions of treatment at 24 months and determine the impact of age and prior surgical procedures on perceptions of efficacy, treatment longevity, and overall satisfaction. In addition, the authors sought to describe the impact of treatment with absorbable suspension sutures on the likelihood a patient will undergo future surgical procedures.

**Methods:**

The first 100 treated patients who underwent treatment with absorbable suspension sutures, by the senior author, were critically evaluated. Subjects completed surveys 24 months following initial treatment.

**Results:**

Of the initial 100 patients, complete records were available for 80 patients (age 39–86). Eighteen (22.5%) received a second treatment with absorbable suspension sutures and average time to second treatment was 23.4 months (range 13–37 months). Overall satisfaction was affected by age, 100% of patients ≤ 50 vs. 60% of patients > 50 (*P* = 0.026). Prior surgery appeared to be a factor in patient perception of efficacy: 82.6% of patients with no prior surgery indicated that absorbable suspension sutures were effective vs. 45.5% of patients with a prior surgical procedure (*P* = 0.0286). Just under one third of pretreatment surgical patients underwent surgery following treatment while 25% of surgery naïve patients went on to have surgery. Importantly, satisfaction with the initial procedure does not preclude later surgery.

**Conclusions:**

Treatment with absorbable suspension sutures is associated with high satisfaction through 24 months and does deter patients from surgery. The combination of lift and volumization results in 4-dimensional rejuvenation that includes rejuvenation of dynamic expression.

**Level of Evidence: 4:**

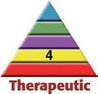

The hallmarks of facial aging include loss of volume, variable skin change, and ptosis of facial and neck anatomy.^1^ Together, these processes give rise to ptotic skin and descent of facial features characteristic of the aging face. Prior to the FDA 510(k) approval of absorbable suspension sutures (Silhouette InstaLift, Sinclair Pharma, Irvine, CA), options for tissue repositioning were limited to surgical interventions. For the aging patient, re-elevation of ptotic tissue is an essential element of facial rejuvenation. A nonsurgical option for tissue repositioning adds an important treatment modality for rejuvenation that may be applied to surgery-naïve patients as well as patients who have already undergone a surgical intervention.

Absorbable suspension sutures (Figure 1) are completely absorbable and are comprised of poly-L-lactic acid (PLLA) and -glycolide (PLGA) copolymers (82% PLLA monomers and 18% PGLA monomers). The suture itself consists of bidirectional cones distributed along the length of a PLLA/PGLA monofilament with intercalated knots. Between the two sets of bidirectional cones (4, 6, or 8 cones per side), there is a 2-cm space in the PLLA/PGLA monofilament. Once the cones are placed in the subcutaneous layer, tissue is advanced over the inferior cones and then elevated by pulling on the superior side of the suture until the inferior tissue is in the desired position. The superior cones are then seated so that the elevated tissue remains in place (Supplementary Video 1). Once seated in place, the superior cones serve as anchors in more fibrous tissues while the inferior cones hold the repositioned tissue in an elevated position. Collagen induced by the PLLA/PGLA within the absorbable suspension suture then encapsulates the device, creating a scaffold that holds the repositioned tissue in place as the suture is absorbed.^2^ The unique lifting capacity of the cones is in large part due to the surface area of the cones, which have 9 times the surface area available for tissue contact than a traditional barb on historical suture applications (Kyungkook Hong, MD, personal communication).

**Figure 1.**
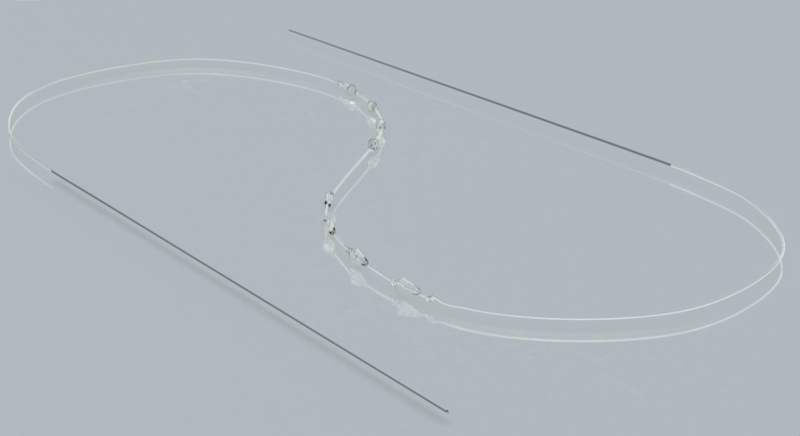
The Silhouette InstaLift microsuspension device is comprised of a PLLA/PLGA polymer containing 82% PLLA and 18% PLGA monomers. The device is entirely absorbable. The bidirectional cones serve to support advanced tissue (distal cones) and anchor repositioned tissue in place (proximal cones). Two 23-gauge, 12-cm needles are appended to the end of the PLLA/PGLA monofilament, and are used to place the device (image reprinted with permission from Sinclair Pharma).

Absorbable suspension sutures have a dual treatment effect. Although the capacity of the cones to lift and reposition tissue is responsible for the immediate outcome of treatment, the collagen-stimulating properties of the resident PLLA/PGLA provide a secondary revolumization that occurs over the course of several months. These mechanisms together are thought to give rise to a global improvement in appearance that has been reported in the context of clinical studies.^2–5^

Since the introduction of absorbable suspension sutures, duration of effect has been of significant interest. Although one study has demonstrated ongoing satisfaction at 12 months, the study population was relatively small, and the 12-month follow-up period is shorter than the duration of effect observed in clinical practice.^5^ Thus, there is a need to better define the longevity of patient satisfaction and clinical effect in a larger cohort. The current study is an extension of a previously published study of 100 patients showing that absorbable suspension sutures are associated with high patient satisfaction and effective treatment of laxity in the mid-face, lower face, and neck at 1 week and 3 months post-treatment.^6^ Here, follow-up data are collected on patient satisfaction and duration of effect up to 24 months.

In addition to the need for more data on long-term patient satisfaction and duration of effect, the question of incorporation into practice has emerged as one important to aesthetic physicians. As part of the current study, investigation into long-term patient satisfaction is dovetailed with an analysis of whether treatment with a nonsurgical modality for tissue repositioning impacts likelihood of patients later seeking surgical interventions. In addition, the analysis explores whether patients who have undergone surgical procedures prior to treatment with absorbable suspension sutures perceive the outcomes of treatment differently than those who have not previously undergone surgery. Within these patterns, the impact of factors such as age and whether the patient went on to receive a second absorbable suspension suture treatment are explored.

## METHODS

### Placement of Absorbable Suspension Sutures

The protocol for placement of absorbable suspension sutures is detailed in the original study of 100 patients on which this report is based.^6^ The method is consistent with that presented in a recent expert consensus paper and is presented here in Supplementary Video 1.^4^ In summary, all treatment adhered to the principles of straight-line vector planning (SLVP),^7^ a method that ensures the lifting capacity of the device is optimized. With the patient in the upright position, the entry and exit points are determined and marked. The inferior exit point is marked 1.5 cm from the “point of action,” or location where the inferiormost cone is needed to support repositioned tissue. For the 8-cone suture, which is most always preferred for the face, the central entry point is then marked 5.5 cm from the exit point along the intended straight-line vector, using the ruler provided within the device package. Finally, the superior exit point is marked 5.5 cm from the entry point. It is not unusual for the superior exit point to be past the hairline.

Prior to placing absorbable suspension sutures, the PLLA/PGLA monofilament must be pulled taut in order to tighten the intercalated knots. With the patient reclined at a 45° angle, the entry and exit points are anesthetized with 1% lidocaine with epinephrine (1:100,000). Importantly, lidocaine should only be injected at the entry and exit points. As long as the suture is advanced in the subcutaneous plane, the patient should not experience pain or discomfort, and any signs of pain are an indication that the suture is in the incorrect plane. Injection of lidocane in the area of the suture tract blunts this important feedback and causes local distension of the tissue. Sedation is not required, as the procedure is well-tolerated. The entry point is dilated with an 18-gauge needle to a depth of 5 mm prior to inserting the needle into the entry point. This facilitates placement of the suture at the proper depth and passage of the cones through the entry point.

The needle appended to the end of the suture should be inserted perpendicular to the skin until the 5-mm depth mark is reached. Taking care to avoid retracting the needle, the needle is turned at a 90° angle into the subcutaneous plane and advanced toward the exit point, maintaining depth in the subcutaneous plane, in a manner that mirrors that when using a cannula. Again, patient discomfort is an indicator that the needle is outside of the correct plane. Once the needle passes through the exit point, the cones are gently pulled though the entry point. This process is then repeated for the other side of the suture. The needle appended to the other side of the suture must be placed in the same entry point in order to avoid a dermal bridge.

Once in place, tissue is advanced over and engaged by the inferior cones. Then, tension is applied to the superior portion of the suture to elevate the tissue slightly past the desired position to allow for the “settling” that can occur over the following days. The superior cones are then anchored by massaging the overlying tissue.

### Patient Population and Survey

Initial treatment for ptotic skin was performed with absorbable suspension sutures in the midface, lower face, and neck: 62% had 8-cone suture placed in the midface, 54% of patients had 8-cone suture placed along the jawline, and 33% of patients had 12-cone absorbable suspension suture treatment in the neck. Patients included in the study were selected based on having been treated in the author’s practice with absorbable suspension sutures. None of the patients had fillers within a year prior to the study. The initial analysis of treatment efficacy and safety in these 100 consecutively treated patients is detailed further in the original study on which this report is based.^6^ Of the 100 original patients treated between December 2015 and November 2016, current and complete records were available for 80 individuals. The remaining 20 patients had moved to other cities or were lost to follow-up. Treatment history was analyzed for these 80 patients to determine the identity of pre- and post-treatment surgical and/or ancillary procedures. All participants returned for physical follow-up and collection of photographs at 24 months. Determination of significance was carried out using the “N-1” chi-squared test for comparison of proportions. All patients paid the usual and customary fee for treatment. Those who completed surveys were offered gift cards. The details of the survey are in the following paragraph.

These 80 patients were asked to complete an 8-question survey (Appendix) aimed at assessing patient experience and overall satisfaction with the procedure at 24 months (mean time to follow-up was 26 months; range, 22–34 months). The electronic survey was administered at the time of the final 24-month follow-up. The patient was provided with an iPad and asked to complete the anonymous survey in a private room. Determination of significance was carried out using the “N-1” chi-squared test for comparison of proportions. This study adhered to the Good Clinical Practice (GCP) and standards set forth in the World Medical Association’s Declaration of Helsinki. Consent for treatment and all included photographs were obtained.

## RESULTS

### Treatment Patterns

Of the 100 patients in the initial study, complete records were available for 80 patients (age range, 39–86 years; 76 females and 4 males). At the time of initial treatment 52 (65%) of these 80 patients had not had a previous surgical intervention, whereas 18 (22.5%) had undergone major surgery, and 10 (12.5%) had undergone an ancillary procedure (Table 1). For patients who had undergone major surgery prior to treatment, the most common procedure was mini facelift (*n* = 8; 10.0%), followed by facelift (*n* = 6; 7.5%) and neck lift/platysmaplasty (*n* = 4; 5.0%). All of the patients who underwent major procedures prior to treatment with absorbable suspension sutures were 50 years of age or older and all patients who underwent ancillary procedures, with the exception of a single patient (age 46), were 50 years of age or older. The most common ancillary procedure was blepharoplasty (*n* = 6; 7.5%), followed by fat injection (*n* = 2; 2.5%), liposuction (*n* = 1; 1.3%), and neckband dissection (*n* = 1; 1.3%).

**Table 1.** Demographics for Patients With Surgery Prior To Treatment With Absorbable Suspension Sutures

Pretreatment surgical procedures (*n* = 28)	
Age, years	
Median	64
Range	53–74
Procedure	No. of patients (%)
None	52 (65)
Major surgery	18 (22.5)
Mini facelift	8 (10)
Facelift	6 (7.5)
Neck lift/platysmaplasty	4 (5.0)
Ancillary procedure	10 (12.5)
Blepharoplasty	6 (7.5)
Other*	4 (5.0)

*Fat injection; liposuction; neckband dissection.

Following treatment with absorbable suspension sutures, 22 of the 80 patients (27.5%; median age 63, range 46–86) underwent surgery (median time to surgery of 19.5 months, range 4 to 41 months) and 13 of these 22 patients (59%) had both surgery and nonsurgical interventions. Of the 58 patients who did not go on to have surgery, 41 (70.7%) received additional nonsurgical treatment such as lasers, fillers, and energy-based skin tightening procedures. Thirteen of the 80 total patients (16.3%) underwent major surgery (Table 2), with the most frequent procedure being neck lift/platysmaplasty (*n* = 9; 11.3%; Table 2), followed by mini facelift (*n* = 3; 3.8%) and facelift (*n* = 1; 1.3%) and 9 (11.3%) underwent an ancillary procedure. Thirteen of these 22 patients (59%) had never undergone a prior surgery while 9 (40.9%) had prior surgery (Table 2). Of note, these 9 patients represent 32.1% of the 28 patients who had undergone a major or ancillary surgical procedure prior to treatment with absorbable suspension sutures. Thus, just under one third of pretreatment surgical patients went on to undergo surgery again following treatment with absorbable suspension sutures and 25% (13/52) of surgery naïve patients went on to have surgery following treatment (7 major surgery; 6 ancillary procedures). Patients who go on to have surgery and patients who do not have similar rates of receiving additional nonsurgical treatment.

**Table 2.** Demographics for Patients Who Went on to Undergo Surgical Procedures Following Treatment With Absorbable Suspension Sutures

Post-treatment surgical procedures (within 2 years of initial treatment) (*n* = 22)	
Age, years	
Median	63
Range	46–86
Procedure	No. of patients (%)
None	58 (72.5)
Major surgery	13 (16.3)
Mini facelift	3 (3.8)
Facelift	1 (1.3)
Neck lift/platysmaplasty	9 (11.3)
Ancillary procedure	9 (8.9)
Fat injections	4 (5.0)
Chin tuck (submental chin excision)	2 (2.5)
Other*	3 (3.8)
Characteristics of post-MST surgical patients (*n* = 22; 27.5%)	
Average time to surgery	19.5 months
Surgical patients with previous surgery	9 (40.9%)

*Neckband dissection; blepharoplasty.

Of the 80 patients evaluated, 18 (22.5%; median age 61.5, range 46-74) received a second treatment with absorbable suspension sutures. The average time to second treatment was 23.4 months (range 13–37 months). Of these 18 patients, 3 (16.7%) went on to later receive fat injections, 1 (5.6%) went on to receive a mini facelift, 1 (5.6%) went on to receive a neck lift/platysmaplasty, and 1 (5.6%) went on to receive a chin tuck/submental skin excision.

These percentages are less than those observed for the overall study population where 27.5% underwent surgery and 67.5% received additional nonsurgical therapy. However, at the time of this writing, patients who received their second treatment with absorbable suspension sutures have done so within the past 12 months. Thus, the average period of time between absorbable suspension suture treatment and surgery has yet to elapse. Most of the 18 patients who received a second absorbable suspension suture treatment also received additional nonsurgical treatment such as fillers, laser, or energy-based therapy (88.8%), a proportion higher than that of patients who did and did not elect to have surgery following treatment with absorbable suspension sutures (59.1% and 70.7%, respectively).

Of the 80 patients treated with absorbable suspension sutures, 13 (16.3%) received touch-up treatment with an average of 2 sutures (one suture per side; range 1–4 total for both sides), a median of 5 months (range 1–11) following initial treatment. These patients represent the earliest patients treated with absorbable suspension sutures. Since that time, adaptations in technique, treatment approach, and a refined understanding of patient selection have negated the need for touch-up treatment before 18 months. Since absorbable suspension sutures were first introduced, this element of “overcorrection,” which can include slight pleating or bunching, along with an understanding that a sufficient number of sutures must be used, has improved outcomes substantially. Currently, the senior author (J.W.F.) may use a single-suture treatment at 18–24 months to prolong the initial results for several months, thus delaying the need for full retreatment. Three (23.1%) of the patients who received touch-up treatment eventually underwent major surgery, a percentage very similar to that for the total study population (23.1% vs. 27.5%). Thus, touch-up treatment with absorbable suspension sutures does not affect the likelihood of future surgery. Five of the 13 touch up patients have received a second silhouette treatment at the time of this publication, a period which represents 42 months of follow-up.

### Survey Results and Impact of Age

Of the 80 patients who were contacted to participate in the satisfaction survey, 34 (42.5%; median age 60 years; range 39–77 years) completed the survey (Appendix). Results are presented in Table 3. Overall, 67.6% of patents could see the results of treatment immediately, 70.6% were satisfied with results, 70.6% found absorbable suspension sutures to be effective treatment at improving age-related change, and 44.1% found the effects of absorbable suspension suture treatment to be long-lasting (ie, lasting longer than 18 months; Table 3). Of note, the neck was most often treated in concert with the midface and/or jawline, and while the jawline and midface may be treated in isolation, they are often treated together. Thus, this study is not sufficiently powered to assess satisfaction based on the combination of areas treated.

**Table 3.** Survey Responses Based on Patient Age

Survey responses	Combined responses	Patients ≤ 50 (*N* = 9)	Patients > 50 (*N* = 25)	
Number of respondents	34/80 (42.5%)	9/34 (26.5%)	25/34 (73.5%)	
Median age, years (range)	59.5 (39-77)	44 (39-49)	64 (53-77)	
Question	Response: yes/true, *n* (%)	Response: yes/true, *n* (%)	Response: yes/true, *n* (%)	Statistical significance, *P*
I found absorbable suture suspension to be tolerable	33 (97)	9 (100)	24 (96)	0.5485
I could see the results of absorbable suture suspension immediately	23 (67.6)	8 (88.9)	15(60)	0.1174
I had manageable discomfort during and after	27 (79.4)	9 (100)	18 (83.3)	0.1972
I had minimal bruising or swelling after absorbable suture suspension	29 (85.3)	9 (100)	20 (80)	0.1524
Overall, I am satisfied with my results	24 (70.6)	9 (100)	16 (64)	0.0386
I would recommend absorbable suture suspension to my family and friends	24 (70.6)	9 (100)	15 (60)	0.0261
I found absorbable suture suspension to be an effective treatment at improving age-related change	24 (70.6)	9 (100)	15 (60)	0.0261
I found the effects of my procedure to be long-lasting (ie, greater than 18 months)	15 (44.1)	6 (66.7)	10 (40)	0.1752

Responses were analyzed to see if age (cutoff, 50 years) significantly affected response. Whether respondents felt absorbable suspension sutures were effective at addressing age-related change was shaped by patient age: All (100%) of patients who were ≤50 years of age indicated that the results were an effective treatment at improving age-related change compared to 64% of patients >50 years of age (*P* = 0.039; 95% CI = 2.19–55.48%).

This pattern was mirrored in patient satisfaction with overall treatment. Although 100% of patients ≤ 50 years of age were satisfied overall with treatment, only 60.0% of patients >50 years of age were satisfied (*P* = 0.026; 95% CI = 5.79–59.26%). Finally, 100% of patients ≤50 years of age reported that they would recommend treatment with absorbable suspension sutures to a friend while 60% of patients >50 years of age would do so (*P* = 0.026; 95% CI = 5.79–59.26%).

### Impact of Prior Surgery

Prior surgery had a significant impact on patient perception of whether absorbable suspension sutures are an effective treatment for improving age-related change. With 82.6% of patients with no prior surgery indicated that absorbable suspension sutures were effective, while 45.5% of the 11 patients who had a surgical procedure before agreed (*P* = 0.0286; 95% CI = 4.03% to 63.4%) (Table 4). In addition to perception of efficacy, overall satisfaction with treatment was higher in the patient group without prior surgery (82.6 vs. 45.5%; *P* = 0.027; 95 CI = 4.35% to 63.4%). Although it is possible that such findings could be somewhat driven by differences in age (median age for patients with prior surgery was 64, range 53–74, vs. 54, range 39–77), more data are needed to complete an analysis that is sufficiently powered to differentiate relative impact of age and surgery. Half (52.2%) of the 23 patients who had not had surgery prior to treatment with absorbable suspension sutures indicated that the results were long-lasting, compared with 27.2% of the 11 patients who had surgery of any kind prior to treatment; however, this difference did not reach significance.

**Table 4.** Survey Responses Based on Whether the Patient Had Prior Surgery

Survey responses	Combined responses	Patients who had surgery prior to treatment^a^	Patients who did not have surgery prior to treatment	
Number of respondents	34/80 (42.5%)	11/34 (32.4%)	23/34 (67.6%)	
Median age (range)	59.5 (39-77)	64 (53-74)	54.0 (39-77)	
Question	Response: yes/true, *n* (%)	Patients who had surgery prior to treatment: yes/true, *n* (%)	Patients who did not have surgery prior to treatment: yes/true, *n* (%)	Statistical significance, *P*
I found absorbable suture suspension to be tolerable.	33 (97.1)	10 (90.1)	23 (100)	0.1308
I could see the results of absorbable suture suspension immediately.	23 (67.6)	5 (45.5)	18 (78.3)	0.0595
I had manageable discomfort during and after.	27 (79.4)	7 (63.6)	20 (87)	0.1198
I had minimal bruising or swelling after absorbable suture suspension.	29 (85.3)	7 (63.6)	22 (95.7)	0.0148
Overall, I am satisfied with my results.	24 (70.6)	5 (45.5)	19 (82.6)	0.0271
I would recommend absorbable suture suspension to my family and friends.	24 (70.6)	6 (54.5)	18 (78.3)	0.1603
I found absorbable suture suspension to be an effective treatment at improving age-related change.	24 (70.6)	5 (45.5)	19 (82.6)	0.0286
I found the effects of my procedure to be long-lasting (ie, greater than 18 months).	15 (44.1)	3 (27.2)	12 (52.2)	0.1760

^a^Includes the subset of survey patients who had surgery (major *n* = 6; ancillary *n* = 5) at some point prior to treatment with absorbable suspension sutures.

All of the 5 survey patients who did not have surgery before treatment with absorbable suspension sutures, but did go on to have surgery after treatment (average age 58.8 years; range, 46–77) indicated that they were satisfied with results of treatment and found the sutures to be an effective treatment at improving age-related change. Just under half (40%) of these patients found the results to be long-lasting (ie, greater than 18 months) and 80% indicated that the results were immediate following treatment. Thus, satisfaction with the initial procedure does not preclude later surgery.

Of the 34 survey responders, 6 (17.6%) received touch-up treatment. For these patients, satisfaction and willingness to recommend treatment to others were not improved by the “touch up.” Rather, in these patients, perceptions of treatment duration (ie, 18 months) were lower (*n* = 0) and absorbable suspension sutures were viewed as an effective treatment for age-related change by 33.3% (*n* = 2) of patients, a percentage far lower than observed for patients overall (70.6%).

In addition to patient perceptions of effect, tolerability and safety were recorded. In the initial study, postprocedure discomfort was managed with acetaminophen only and narcotics were unnecessary. In this 24-month follow-up study, there were no late-onset adverse events reported. Of note, the neck was most often treated in concert with the midface and/or jawline, and while the jawline and midface may be treated in isolation, they are often treated together. Thus, this study is not sufficiently powered to assess satisfaction based on the combination of areas treated.

**Figure 2.**
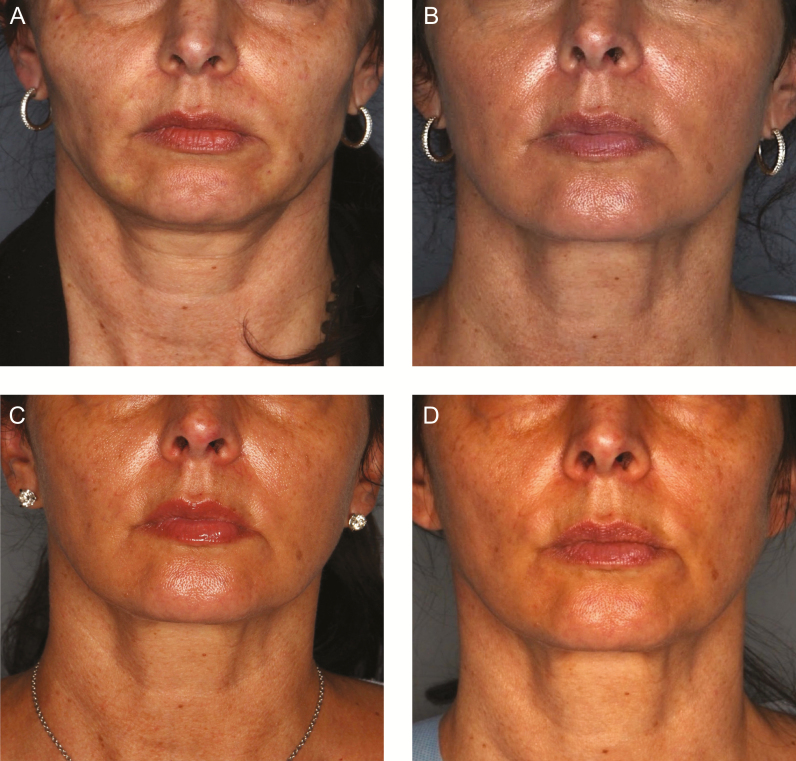

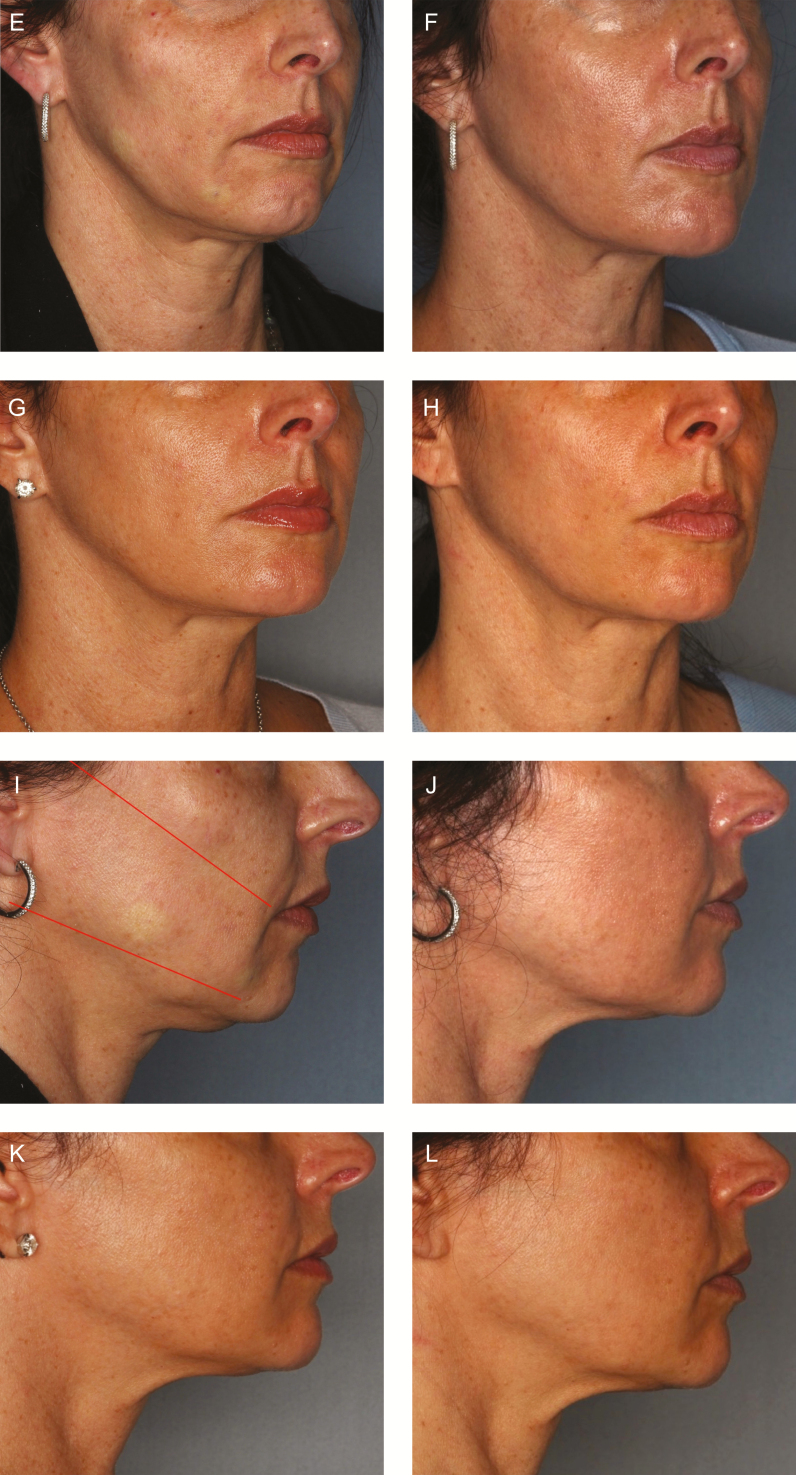
A 48-year-old female with moderate midfacial volume deficit with ptosis, moderate jowling, and submental laxity underwent placement of 2 sets of 8-cone sutures per side; one placed along the midface (nasolabial fold to temporal sideburn) in a vertical vector and the other placed along the jawline (marionette line to temporal sideburn) in a vertical vector. Follow-up photographs were taken at 6, 18, and 24 months (before second Silhouette procedure). (A) Preprocedure frontal view illustrating midfacial volume deficit and loss of inverted triangle of youth. (B) 6-month follow-up frontal view illustrating improved midfacial volume, improvement in midface ptosis, and reduction in submental laxity. (C) 18-month follow-up showing sustained improvement in midfacial volume, midface ptosis, and submental laxity compared to baseline. (D) 24-month follow-up prior to second Silhouette procedure displaying a continued improvement in overall facial appearance; second procedure performed with two sets of 8-cone sutures on each side, same location as first procedure, to augment results. (E) Preprocedure right three-quarter view illustrating moderate jowling. (F) 6-month follow-up right three-quarter view illustrating improved streamline of the jaw with jowl reduction. (G)18- and (H) 24-month follow-up right three-quarter view demonstrating continued improved streamline of the jaw with jowl reduction as compared to baseline. (I) Preprocedure right profile view illustrating moderate submental laxity with suture placement indicated. (J) 6-month follow-up right profile view displaying improved submental laxity and tighter cervical-mental angle. (K) 18- and (L) 24-month follow-up right profile views demonstrating sustained improvement in submental laxity and tighter cervical-mental angle. The patient was also treated with neuromodulators in the glabella, crow’s feet, upper forehead, and anterior hairline.

**Figure 3.**
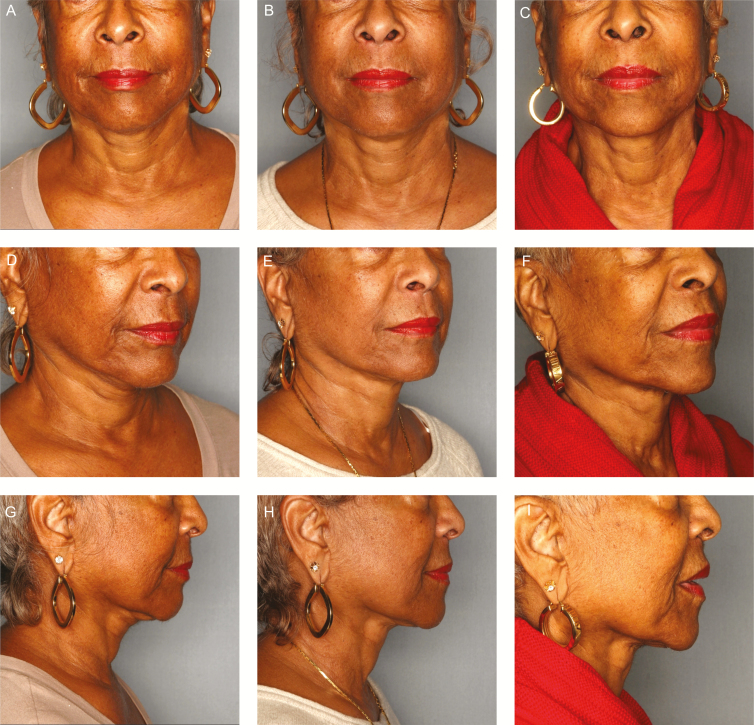
A 70-year-old female with history of mini facelift in 10 years before current treatment with mild midfacial volume deficit with ptosis, moderate jowling, and submental laxity underwent placement of 2 sets of 8-cone sutures per side of the midface and 1 set of 12-cone sutures per side of the neck. Two 8-cone sutures were placed superolaterally along the midface (nasolabial fold to temporal sideburn) in a vertical vector. One 12-cone suture placed on each side of the neck (submentum to mastoid fascia) in a vertical vector. Follow-up photographs were taken at 3 and 28 months. (A) Preprocedure frontal view illustrating midfacial volume deficit and loss of inverted triangle of youth. (B) 3-month follow-up frontal view illustrating improved midfacial volume, reduction of indentation of nasolabial folds, improvement in midface ptosis, and reduction in submental laxity. (C) 28-month follow-up showing sustained improvement in midfacial volume and midface ptosis as compared to baseline. (D) Preprocedure right three-quarter view illustrating moderate jowling and submental laxity. (E) 3-month follow-up right three-quarter view illustrating improved streamline of the jaw with jowl reduction. (F) 28-month follow-up right three-quarter view demonstrating continued improved streamline of the jaw with jowl reduction as compared to baseline. (G) Preprocedure right profile view illustrating moderate submental laxity with suture placement indicated. (H) 3-month follow-up right profile view displaying improved submental laxity and tighter cervical-mental angle. (I) 28-month follow-up right profile view demonstrating sustained improvement in submental laxity and tighter cervical-mental angle.

**Figure 4.**
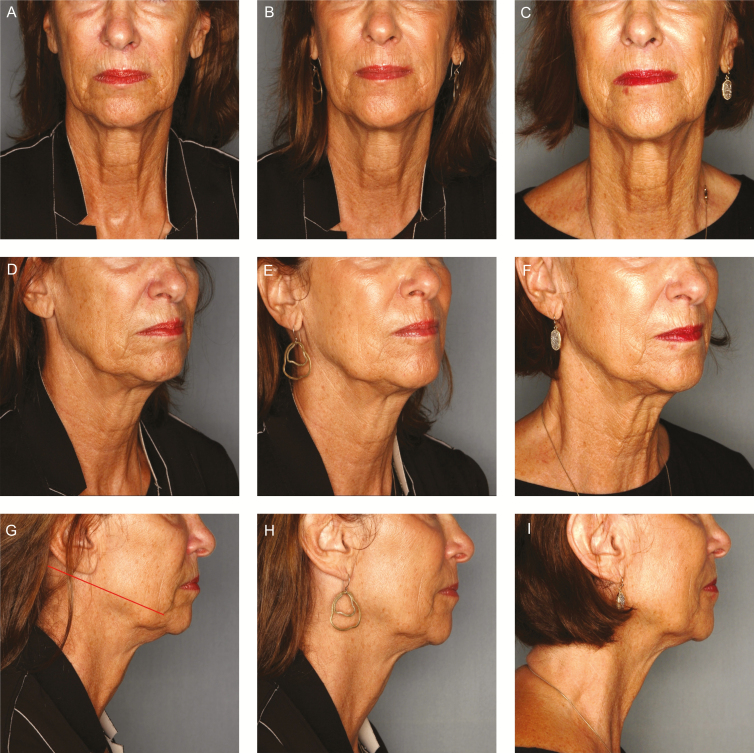
A 64-year-old female with severe midfacial volume deficit with ptosis, moderate jowling, and submental laxity who underwent placement of 2 sets of 8-cone sutures per side. Two 8-cone sutures placed along the jawline (marionette line to temporal sideburn) in a vertical vector. Follow-up photographs were taken at 3 and 26 months. (A) Preprocedure frontal view illustrating midfacial volume deficit and loss of inverted triangle of youth. (B) 3-month follow-up frontal view illustrating improved midfacial volume, reduction of indentation of nasolabial folds, improvement in midface ptosis, and reduction in submental laxity. (C) 26-month follow-up showing sustained improvement in midfacial volume, reduction of indentation of nasolabial folds, improved midface ptosis, and submental laxity compared to baseline. (D) Preprocedure right three-quarter view illustrating moderate jowling. (E) 3-month follow-up right three-quarter view illustrating improved streamline of the jaw with jowl reduction. (F) 26-month follow-up right three-quarter view demonstrating continued improved streamline of the jaw with jowl reduction when compared with baseline. (G) Preprocedure right profile view illustrating moderate submental laxity with suture placement indicated. (H) 3-month follow-up right profile view displaying improved submental laxity and tighter cervical-mental angle. (I) 26-month follow-up right profile view demonstrating sustained improvement in submental laxity and tighter cervical-mental angle. The treatment history of the patient includes energy-based tightening to the face and neck over 1 year prior to treatment.

Figures 2-4 are representative patients treated with absorbable suspension sutures for the correction of ptosis and segmental laxity. The results shown illustrate the nature of results at 24 months post-treatment.

## DISCUSSION

Patient desire to avoid downtime and the demand for nonsurgical interventions is increasing. Prior to the introduction of absorbable suspension sutures, the descent of facial features that occurs as a result of aging was treatable only with rhytidectomy. With the introduction of any new technology comes the need to fully characterize both the nature and duration of results as well as patient perceptions of and satisfaction with treatment. In addition, any new technology must fit well within the current treatment armamentarium. In surgical practices, in particular, an understanding of the impact of prior surgical experiences on noninvasive repositioning as well as the impact of nonsurgical repositioning on future surgical procedures is central to the application of the technology.

In the earliest clinical studies of absorbable suspension sutures, Nestor et al made the interesting observation that while patient satisfaction with multiple FACE-Q domains and perception of age improved through the end of the 12-month study, this improvement was independent of measured outward lift.^5^ This study was the first to provide data on what long-time users of absorbable suspension sutures had already observed: clinically, absorbable suspension sutures, in the appropriately selected patient, are associated with high satisfaction for upwards of 18–4 months. In the author’s (J.W.F.) experience, at 24 months, the treatment is nearing the end of aesthetic effect, and patients often elect to undergo additional treatment or to receive surgery. In the current study, the authors sought to better characterize the satisfaction at 24 months, as well as to place absorbable suspension sutures into context for the surgical practice.

In our 100-patient cohort, the average time to retreatment was 23.4 months (range, 13–37 months) and average time to surgery, for those electing to transition to the more invasive option, was 19.5 months (range, 4–41 months), indicating a significant duration of effect in well-selected patients. At 24 months post-treatment, a majority of patients (67.6%) reported that they could see the results of treatment immediately, and an even greater percentage (70.6%) found absorbable suspension sutures to be an effective treatment for improving age-related change. Perception of whether absorbable suspension sutures are an effective treatment for improving age-related change was affected by age (77% of patients 60 years of age and under and 100% of patients 50 years of age and younger). For overall satisfaction, 100% of patients ≤ 50 are satisfied with absorbable suspension suture treatment and 60% of patients >50 were satisfied with absorbable suspension suture treatment. Finally, 100% of patients under 50 would recommend treatment to a friend, compared with 70.6% overall and 60% of patients over the age of 50. Age was not a determinant of whether the patient felt the results were immediately apparent or found absorbable suspension suture treatment to be long lasting (ie, lasting longer than 18 months). Together, these results indicate that most often, patients under 50 years old are happiest with the results of absorbable suspension sutures. That does not mean that age in and of itself is a determinant of satisfaction, especially in the appropriately selected patient, but rather that patient characteristics and expectations should be carefully considered in more mature patients. In a recent expert consensus paper, patient selection is discussed extensively, as are atypical patient groups such as more mature patients.^4^ In addition, the authors (J.W.F.) have found that treatment with absorbable suspension sutures is especially suitable for younger patients who wish to avoid downtime or who would like to avoid the scar that accompanies surgery (in particular patients with skin of color and men with patterns of hair growth that do not permit effective concealment of scars).

Absorbable suspension sutures can be used for tissue repositioning in patients who have already had surgery to maintain results, patients who have had nonfacelift surgery who wish to delay surgery, or those who do not have experience with surgical outcomes. Within the study population, 18 (22.5%) had undergone major surgery, and 10 (12.5%) had undergone an ancillary procedure. These patients were less likely to view absorbable suspension sutures as an effective treatment at improving age-related change (45.5% vs. 70.6% overall and 82.6% of patients who did not have surgery prior to treatment), and less likely to be satisfied overall with the procedure (45.5% vs. 70.6% overall and 82.6% of patients who did not have surgery prior to treatment). We hypothesize that this difference is due to patients’ previous experience with surgical results, but recognize that age could be a lurking variable, in particular because the median age for the surgical group is 10 years higher than the nonsurgical group (median, 64 years; and range, 53–74 years; vs. median, 54 years; and range, 39–77 years, respectively; Table 4). In order to understand the relative impact of age and prior surgery on perception of absorbable suspension suture treatment efficacy, a larger data set is needed so that patients in each group can be matched for age and prior procedures and subset analysis is sufficiently powered. Overall, older patients and/or patients who have had previous surgery are less likely to perceive the treatment as effective.

As one may expect, nonsurgical repositioning with absorbable suspension sutures is not a surrogate for surgery. Even so, we were interested in understanding whether patients treated with absorbable suspension sutures were more or less likely to go on to receive surgical procedures. Following treatment with absorbable suspension sutures, 22 patients (27.5%; median age 63, range 46–86) underwent surgery (median time to surgery is 19.5 months) and 54 (67.5%) received additional nonsurgical therapy. Of these 22 patients, 9 (40.9%) had undergone surgery prior to absorbable suspension sutures and 13 (59%) had not. Thus, patients who have had surgery prior to treatment with absorbable suspension sutures often go on to have additional surgical procedures (40.9% of patients with prior surgery went on to have additional surgery).

These data presented are in agreement with the authors’ clinical experience: treatment with absorbable suspension sutures does not cause patients to delay or forgo surgical procedures. Rather, treatment allows patients without prior surgical procedures a glimpse at the importance of repositioning and opens the door to more comprehensive rejuvenation and surgical procedures in the face and neck. Absorbable suspension sutures allow patients to see how repositioning can affect their appearance without invasive surgery. Indeed, in this data set, many of the patients who went on to have facelifts were initially against to the idea of surgery at the time of initial treatment with absorbable suspension sutures.

Although this study does lend insight into patient perceptions of efficacy and longevity, there are limitations to this study. First, the 2-year follow-up was conducted with the first 100 consecutive patients treated in the author’s (J.W.F.) practice. Thus, the outcomes only partially represent what can be achieved with optimal technique that has been developed since absorbable suspension sutures were first introduced. In particular, successful and durable treatment requires what can be interpreted as “overcorrection.” Tissue that is repositioned can settle over the following days. Thus, the ideal degree of repositioning has evolved to be more aggressive. Although too superficial placement can cause dimpling from the cones catching on the dermis, pleating and bunching of tissue is a normal part of treatment sequelae and can be expected to dissipate within 3–5 days. In addition, treatment requires a sufficient number of sutures, most often 3 per side in the midface. These two factors in particular are linked to the degree of improvement over time and the durability of results. In addition, in order to understand the relative contributions of age and prior surgery to satisfaction with treatment and longevity of effect, a larger data set is needed. Finally, to better understand the nature of the results, future studies could include additional questions from validated scales (eg, FACE-Q domains). The authors recognize that some of the questions in the survey (Appendix) are subject to recall bias. Thus, no attempt is made here to tie initial impressions to long-term outcomes. Importantly, however, perceptions of the treatment as effective at 24 months is based on perception at the time of questioning.

The place of absorbable suspension sutures in aesthetic practice can be informed by the understanding that repositioning is a central part of a balanced approach to rejuvenation. Prior to the introduction of absorbable suspension sutures, nonsurgical “lifting” was restricted to what could be achieved using fillers. This absence led to an overuse of fillers as the only nonsurgical option to “raise” descended facial features. Although composite lifting is a phenomenon that should be artistically leveraged, the over use of fillers when the true need is one of repositioning leads to an “overfilled” and artificial appearance.

Overfilling can manifest as a blatant exaggeration of facial volume, but can also be more insidious. In the overfilled patient who appears appropriately managed at rest, dynamic facial expression and movement is not youthful, but distorted by excessive revolumization. This concept is a foundational observation that gave rise to the guiding principles of the aesthetic theory of Inverso.^8^ Although the concept of the inverted triangle of youth explains age-related differences observed at rest, Inverso takes into account the need to preserve and restore youthful dynamics in expression as well as facial proportion. Filling the tissues when the primary need is repositioning threatens the natural facial movements that guide our innate perceptions of youth and beauty just as much, if not more than the proportions of a static expression. In particular, we have found in subsequent patients that the use of neuromodulation of the depressor anguli oris in combination with filler to the marionette and restoration of lost volume to the midface leads to the desired inverted triangle of youth. This will disable the primary retractor, the depressor anguli oris, allowing upper facial musculature and absorbable suture resuspension to lift the lower face in addition to the midface. Another reason the senior author prefers to avoid over volumizing as a way to lift the face is the resultant distortion of normal facial expression and the impairment of normal expressivity over short time intervals of daily conversations with friends, family, and the general public. Importantly, in the author’s experience, absorbable suspension sutures do not affect the dynamic function of facial musculature.

Since the introduction of absorbable suspension sutures, the authors have consistently observed high ongoing patient satisfaction in the absence of significant revolumization and surgical-scale repositioning. It is our hypothesis that absorbable suspension sutures satisfy the tenants of an Inverso-guided approach to 4-dimensional rejuvenation. In many instances, static photographs showing patients before and after treatment highlight an apparent disconnect between the degree of volume restoration present and the high satisfaction associated with the treatment. Although it is tempting to categorize nonsurgical results as “subtle,” we would argue that much of the observed satisfaction is rooted in a significant rejuvenation of dynamic expression achieved through the dual action of repositioning and revolumization achieved with absorbable suspension sutures. Further clinical and patient-reported observations of improvement in skin quality support this 4-dimensional rejuvenation.

With the theory of Inverso in mind, absorbable suspension sutures may be combined with other nonsurgical modalities such as fillers, energy-based skin tightening, and treatment with botulinum toxin, among others.^9^ Layering and multimodal treatment can further improve appearance and allow for a natural and rejuvenated appearance. Indeed, within our data set, a majority of patients treated with absorbable suspension sutures also underwent additional nonsurgical treatment.

## CONCLUSIONS

Microsuspension technology is the only nonsurgical product that can effectively reposition tissue. Treatment is associated with high satisfaction and duration through 24 months and does not eclipse patient desire for surgical procedures. Rather, the opposite appears to be the case. The combination of lift and volumization leads to a 4-dimensional rejuvenation that includes rejuvenation of dynamic expression. Together, these features make absorbable suspension sutures an important element of aesthetic practice.

## Supplementary Material

This article contains supplementary material located online at http://www.asjopenforum.com.

ojz029_suppl_Supplementary_Appendix_A
