# Supplementary material for: Nonsurgical Tissue Repositioning: Analysis of Long-Term Results and Patient Satisfaction From 100 Absorbable Suture Suspension Cases
Source: Aesthet Surg J Open Forum. 2019 Oct 19;2(1):ojz029. doi: 10.1093/asjof/ojz029 (PMC7780437; doi:10.1093/asjof/ojz029)
Supplement: ojz029_suppl_Supplementary_Appendix_A [file ojz029_suppl_supplementary_appendix_a.pdf]

Name: ___________________________ Date: _________________

Silhouette 2-Year Study Survey

1. I found absorbable suture suspension to be tolerable. (T/F)
2. I could see the results of absorbable suture suspension immediately. (T/F)
3. I had manageable discomfort (no medication required to make better) during and after absorbable suture suspension. (T/F)
4. I had minimal bruising or swelling after absorbable suture suspension. (T/F)
5. Overall, I am satisfied with my results from absorbable suture suspension. (T/F)
6. I would recommend absorbable suture suspension to family and friends. (T/F)
7. I found absorbable suture suspension to be an effective treatment at improving age-related change. (T/F)
8. I found the effects of my procedure to be long-lasting (i.e. lasting longer than 18 months). (T/F)
